# Vasa previa with bipartite placenta and velamentous cord insertion

**DOI:** 10.1016/j.radcr.2026.05.038

**Published:** 2026-06-03

**Authors:** Montacer Hafsi, Catherine R. Duvinage, Serge Sassine, Youssef Hamdan, Hala Hassan, Samer Maalouf, Francois Devianne

**Affiliations:** Department of Gynecology and Obstetrics, Paris Saclay-North Essonne Hospital Group, 1 Parvis de l'hopital, Orsay 91400, France

**Keywords:** Vasa previa, Bipartite placenta, Velamentous cord insertion, Prenatal diagnosis, Cesarean section, Benckiser hemorrhage, Color doppler ultrasound

## Abstract

Vasa previa is a rare but potentially life-threatening obstetric condition characterized by fetal vessels traversing the membranes over or near the internal cervical os. Without prenatal diagnosis, it carries a perinatal mortality rate exceeding 50% due to the risk of Benckiser hemorrhage upon membrane rupture. We present the case of a 24-year-old primigravida diagnosed with vasa previa associated with a bipartite placenta and velamentous cord insertion at 32 weeks of gestation. The diagnosis was initially made at the referring hospital in Bordeaux and confirmed at our institution using transvaginal ultrasound with color Doppler imaging. The patient underwent a planned cesarean delivery at 36 weeks and 5 days, resulting in a healthy female neonate weighing 2600g with excellent Apgar scores (9/10/10). This case highlights the critical importance of prenatal diagnosis of vasa previa and the excellent outcomes achievable with timely cesarean delivery before the onset of labor. Transvaginal ultrasound with color Doppler remains the gold standard for detection in high-risk pregnancies.

## Introduction

Vasa previa is defined as a condition in which fetal vessels, unprotected by placental tissue or umbilical cord, traverse the membranes over or within 2 cm of the internal cervical os [1,2]. This rare obstetric complication occurs in approximately 1 in 1200 to 1 in 5000 pregnancies, with recent studies suggesting an incidence of 0.46 per 1000 deliveries [3,4]. The condition carries significant perinatal morbidity and mortality when undiagnosed, as rupture of these vessels during labor or membrane rupture can lead to rapid fetal exsanguination—a catastrophic event known as Benckiser hemorrhage [5,6].

Vasa previa is classified into 3 types: Type I occurs with velamentous cord insertion where the umbilical cord inserts into the membranes rather than the placenta; Type II involves fetal vessels connecting lobes of a bilobed or succenturiate placenta; and the recently described Type III features aberrant vessels extending from the placenta through the membranes in a ``boomerang'' pattern without velamentous insertion or multilobed placenta [7,8]. Risk factors include low-lying placenta or placenta previa, velamentous cord insertion, bilobed or succenturiate placenta, in vitro fertilization, and multiple gestations [9].

The advent of prenatal diagnosis using transvaginal ultrasound with color Doppler has dramatically improved outcomes. Historical data reported perinatal mortality rates of 58% before the ultrasound era [10]. Contemporary studies demonstrate that prenatal diagnosis with planned cesarean delivery before labor yields survival rates exceeding 97%, compared to only 72% in undiagnosed cases, representing a 25-fold reduction in perinatal death [11,12]. We present a case of vasa previa with bipartite placenta successfully managed through prenatal diagnosis and planned cesarean delivery.

## Case presentation

A 24-year-old primigravida (G1P0) presented to our institution at 33 weeks and 6 days of gestation for establishment of care following relocation from Bordeaux, France. Her pregnancy had been uncomplicated, conceived spontaneously. The patient's medical history was unremarkable with no surgical, medical, or family history of note. She had no known allergies and was not taking any medications. Her prepregnancy weight was 69 kg with a height of 180 cm (BMI 21.3 kg/m²).

**Fig. 1:**
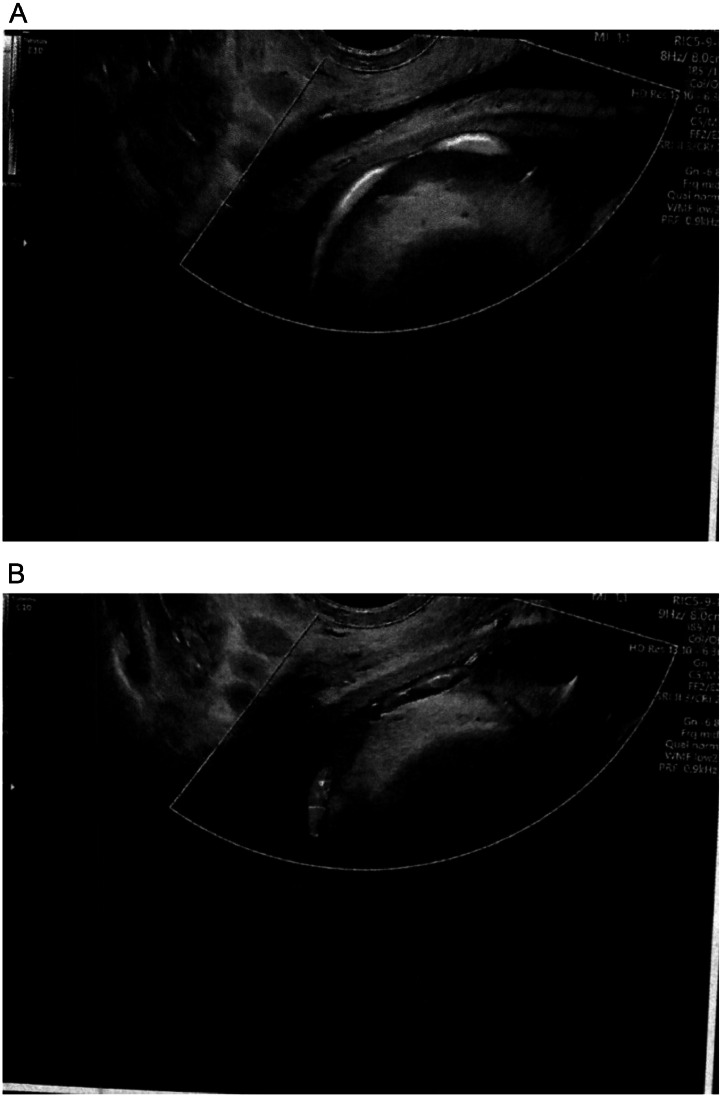

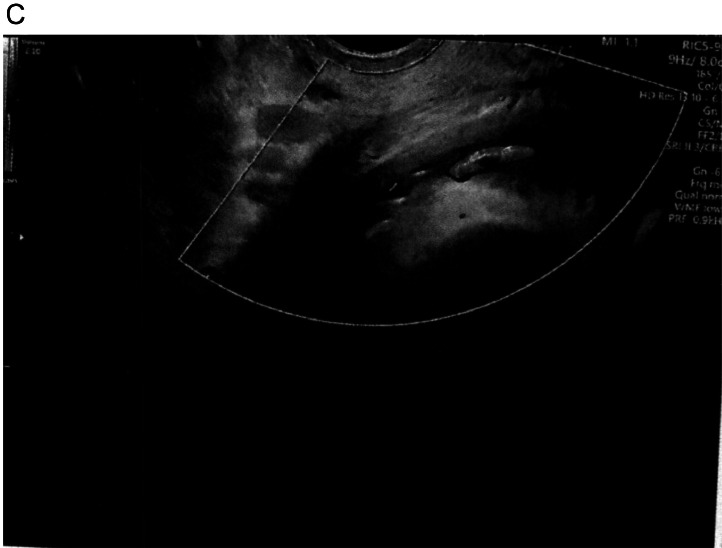
(A) Transvaginal grayscale ultrasound at 32 weeks demonstrating the cervix with vasa previa vessels visible as tubular anechoic structures traversing the membranes overlying the internal cervical os. (B) Transvaginal ultrasound demonstrating the velamentous vessels as linear hypoechoic structures coursing through the lower uterine segment membranes in close proximity to the internal os. The cervical canal is clearly visualized and (C) Additional transvaginal ultrasound view showing the relationship between the vasa previa vessels and the cervix. The unprotected fetal vessels are identified crossing the region of the internal cervical os, confirming the diagnosis prior to color Doppler confirmation.

Prenatal screening had been appropriately performed, with first-trimester combined screening showing a low risk for trisomy 21 (1/9500). The second-trimester morphology ultrasound at 22 weeks demonstrated normal fetal anatomy with a low-lying anterior placenta. A third-trimester ultrasound at 32 weeks and 2 days, performed at the CHU de Bordeaux, revealed a bipartite placenta with velamentous cord insertion and a vasa previa vessel passing in close proximity to the cervix in the right laterocervical region (Figs. 1A–C). The grayscale images clearly demonstrated the tubular vascular structures coursing through the membranes overlying the internal cervical os. The patient was appropriately counseled about the risk of Benckiser hemorrhage and the indication for cesarean delivery between 36 and 37 weeks.

Upon initial evaluation at our institution, the patient was clinically well with blood pressure of 109/72 mmHg and pulse of 70 bpm. Physical examination revealed a fundal height of 33 cm, fetal heart tones present, and cephalic presentation. Urinalysis showed trace protein (30 mg/dL). Laboratory investigations including complete blood count (hemoglobin 11.7 g/dL, platelets 300 G/L), coagulation studies (PT 100%, aPTT ratio 1.04, fibrinogen 4.49 g/L), blood type and screen (O positive, antibody screen negative), and toxoplasmosis serology (negative) were all reassuring. Group B streptococcus screening was negative.

**Fig. 2:**
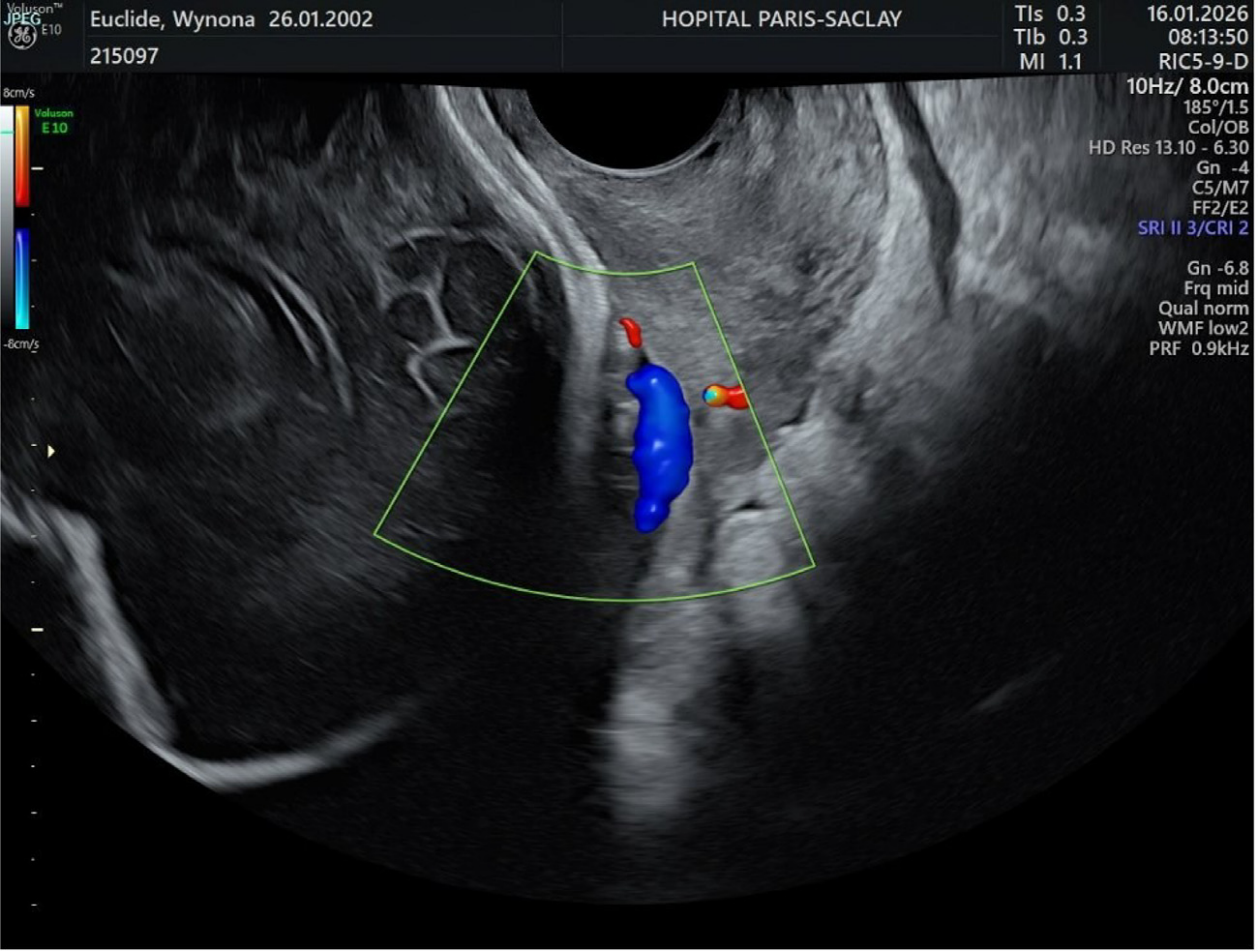
Transvaginal ultrasound with color Doppler imaging at 34 weeks and 6 days confirming vasa previa. The image shows fetal vessels with characteristic arterial (red) and venous (blue) flow patterns crossing directly over the internal cervical os. This color Doppler appearance provides definitive confirmation of vasa previa with the vessels at high risk of rupture during labor or membrane rupture.

A confirmatory ultrasound examination at 34 weeks and 6 days was performed using transabdominal and transvaginal approaches with color Doppler imaging on a Voluson E10 ultrasound system. This confirmed a singleton pregnancy in cephalic presentation with appropriate fetal biometry (estimated fetal weight 2116g, 12th percentile). The examination confirmed a bipartite placenta with an anterior portion distant from the cervix and a posterior portion more than 40 mm from the internal os, with velamentous cord insertion. Critically, transvaginal color Doppler imaging clearly demonstrated vasa previa vessels overlying the internal cervical os (Fig. 1, Fig. 2), with characteristic arterial and venous flow patterns. Umbilical artery Doppler indices were normal (pulsatility index 0.99, 81st percentile). Based on these findings, a scheduled cesarean delivery was planned for 36 weeks and 5 days.

Preoperative evaluation included anesthesia consultation and echocardiography (requested due to early pregnancy dyspnea, which was unremarkable). The patient was hospitalized the day before surgery at 36 weeks and 4 days. On January 29, 2026, a planned cesarean delivery was performed under spinal anesthesia. Careful attention was paid during uterine incision to avoid the aberrant vessels. Manual removal of the placenta was required (artificial delivery). A female infant was delivered weighing 2,600g (35th percentile) with Apgar scores of 9, 10, and 10 at 1, 5, and 10 minutes respectively, requiring no resuscitation.

**Fig. 3:**
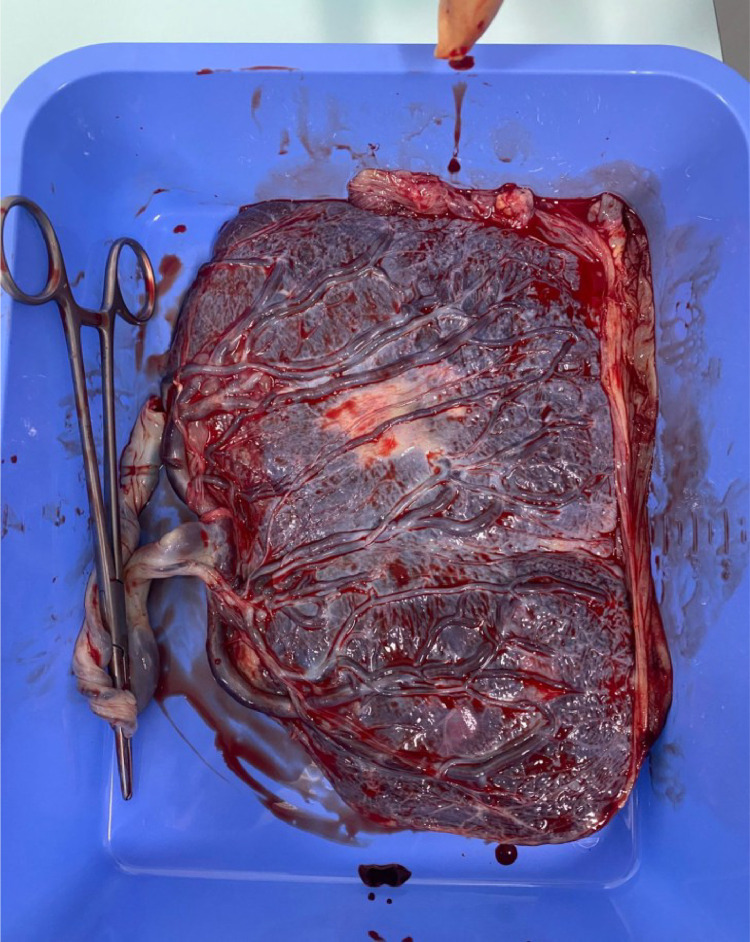
Fetal surface of the bipartite placenta demonstrating velamentous cord insertion with fetal vessels traversing the membranes. The umbilical cord is clamped with Kelly forceps at the site of membrane insertion. The branching chorionic vessels are clearly visible across the placental surface.

**Fig. 4:**
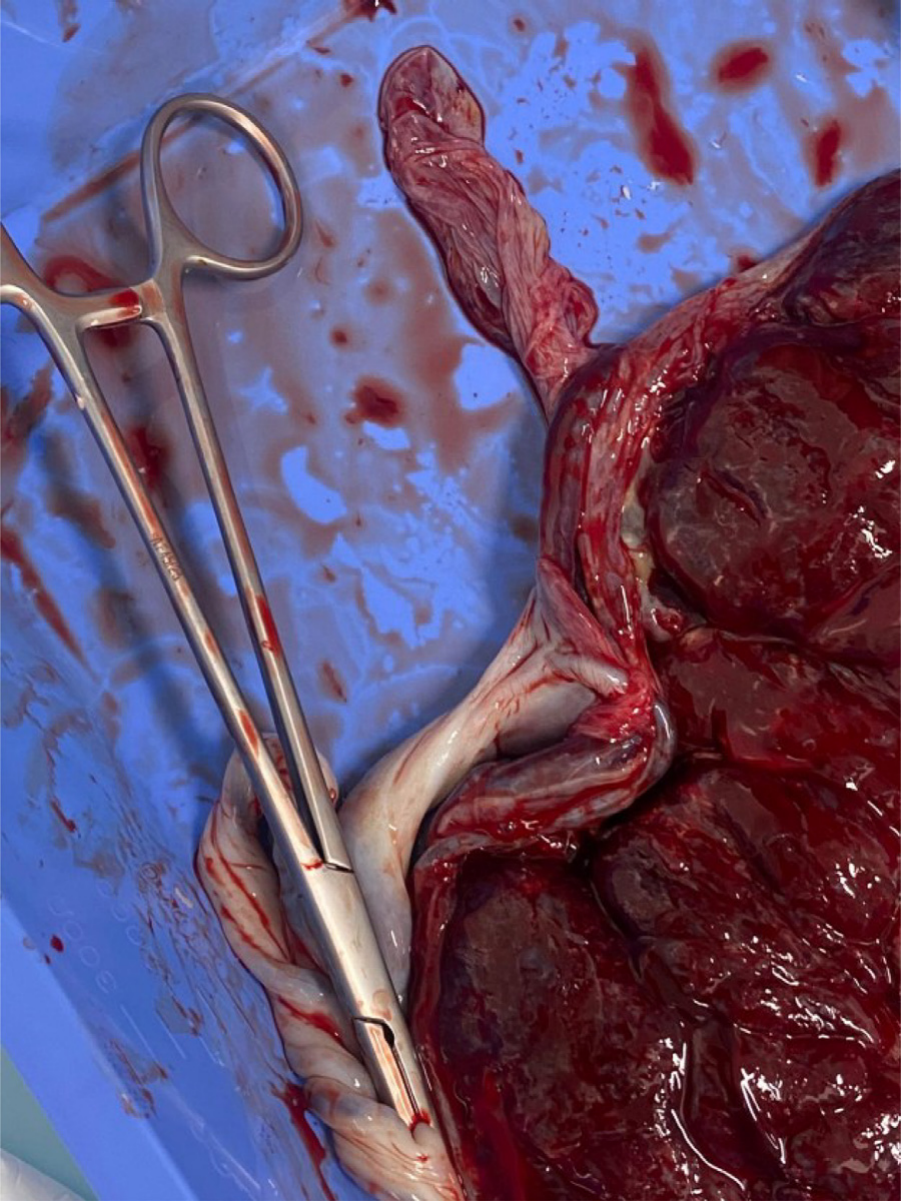
Close-up view of the velamentous cord insertion showing the umbilical vessels (2 arteries and 1 vein) transitioning from the cord to traverse through the membranes before reaching the placental tissue. Note the lack of Wharton's jelly protection as the vessels course through the membranes.

**Fig. 5:**
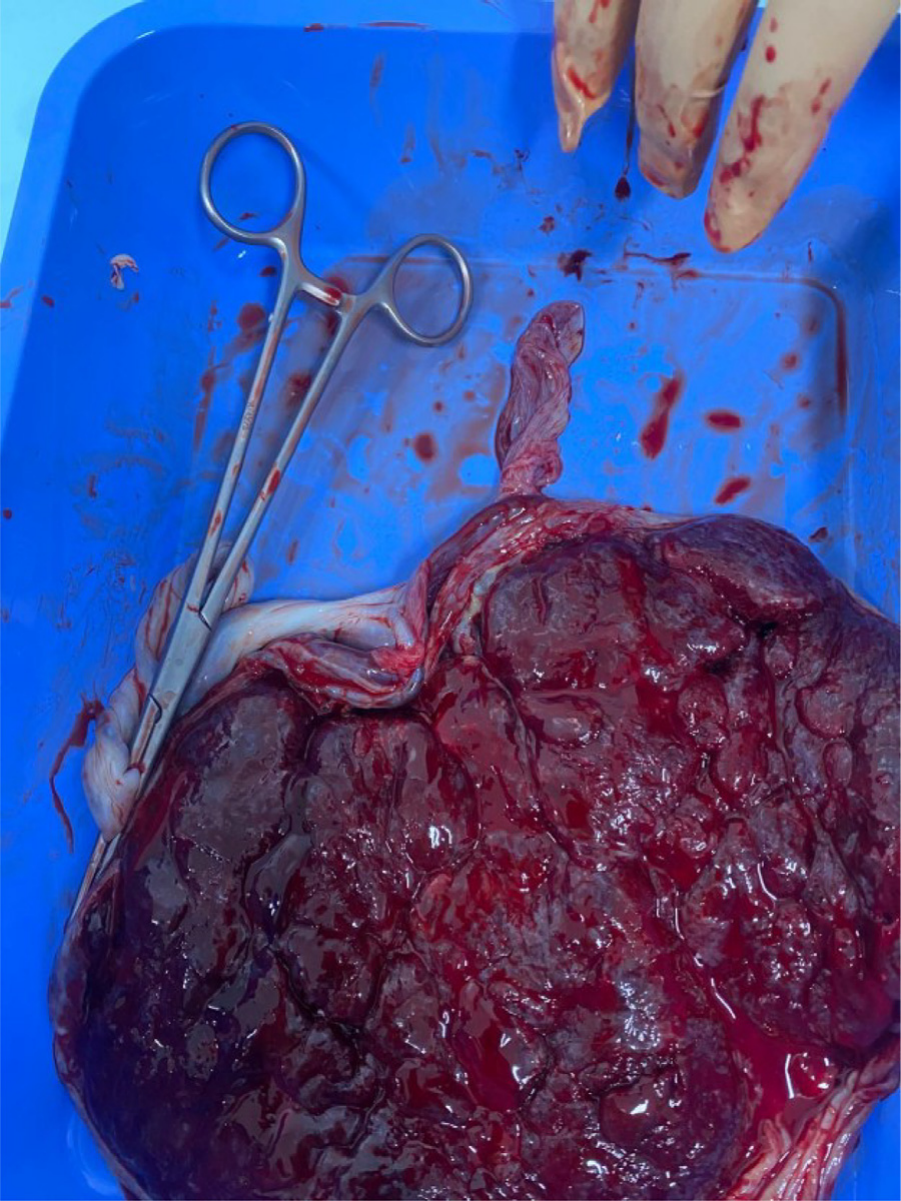
Maternal surface of the bipartite placenta showing the characteristic lobulated appearance. The placenta consists of 2 distinct lobes connected by membranes containing fetal vessels.

**Fig. 6:**
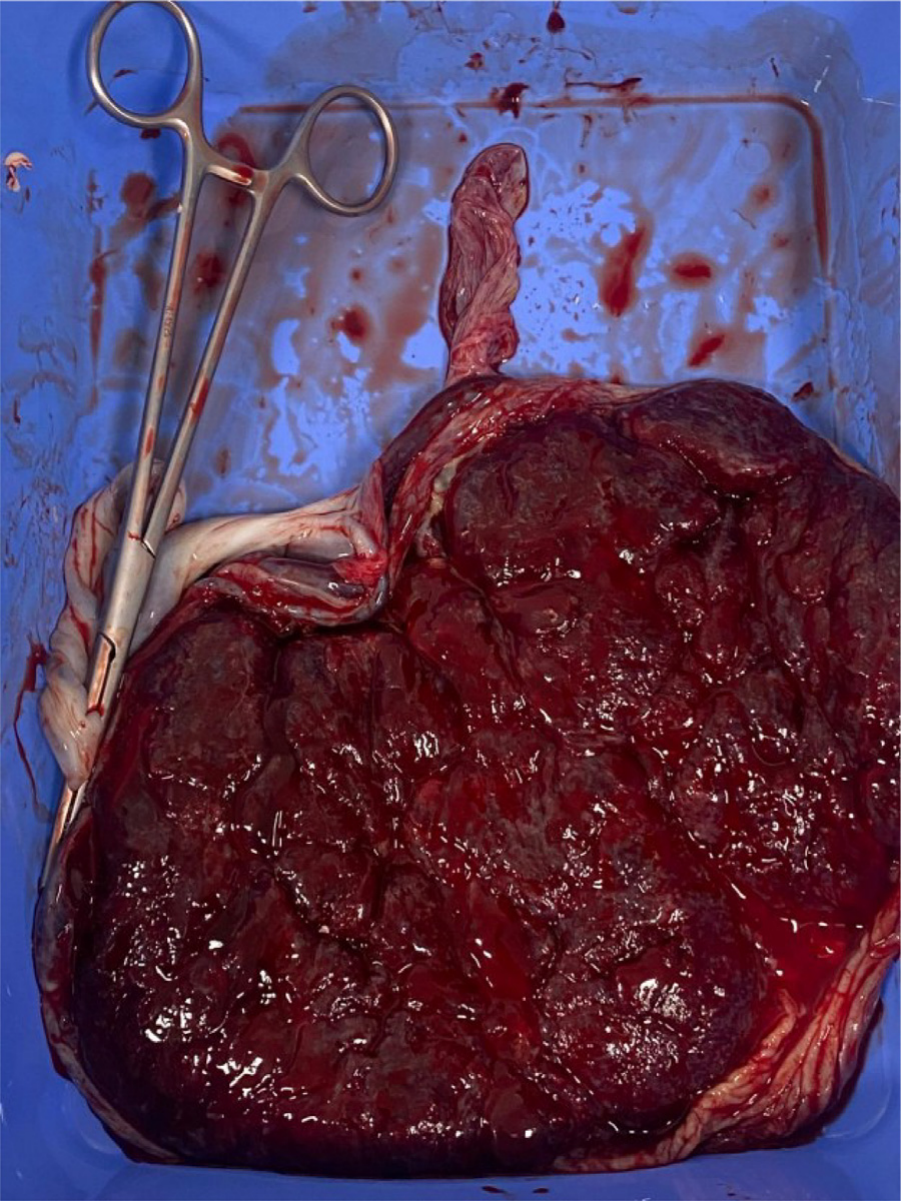
Alternative view of the maternal surface demonstrating the complete bipartite placenta with both lobes visible. The cord insertion site with velamentous vessels is evident at the superior aspect of the specimen.

Gross examination of the placenta confirmed the diagnosis of bipartite placenta with velamentous cord insertion (Fig. 3, Fig. 4, Fig. 5, Fig. 6). The fetal surface demonstrated the characteristic branching pattern of chorionic vessels, while examination of the cord insertion site revealed the velamentous configuration with vessels traversing the membranes before reaching the placental tissue. The maternal surface showed 2 distinct placental lobes. Both mother and infant had an uncomplicated postoperative course Fig. 4, Fig. 5.

## Discussion

This case demonstrates the successful prenatal diagnosis and management of vasa previa associated with bipartite placenta and velamentous cord insertion. The combination of these placental anomalies represents a classic presentation of Type II vasa previa, where fetal vessels course through the membranes connecting separate placental lobes [1,7]. Our patient exhibited multiple risk factors including bilobed placenta and velamentous cord insertion, which together account for the majority of vasa previa cases [13].

The critical importance of prenatal diagnosis cannot be overstated. A systematic review by Zhang et al. [11] demonstrated that prenatal diagnosis of vasa previa is associated with total and intact perinatal survival rates of 99% and 97%, respectively, compared to only 72% and 28% in undiagnosed cases. This represents a 25-fold increased risk of perinatal death when the diagnosis is missed. Our case exemplifies the excellent outcomes achievable when vasa previa is diagnosed prenatally and managed with planned cesarean delivery before the onset of labor.

The sonographic diagnosis of vasa previa relies on both grayscale and color Doppler imaging modalities. As demonstrated in Figures 1A-C, grayscale transvaginal ultrasound can identify vasa previa vessels as tubular anechoic or hypoechoic structures traversing the membranes over the cervix. These vessels appear as linear structures coursing through the lower uterine segment in close proximity to the internal os. However, color Doppler imaging (Fig. 2) provides definitive confirmation by demonstrating characteristic arterial and venous flow within these structures [14]. The combination of grayscale imaging for anatomical assessment and color Doppler for flow confirmation represents the gold standard diagnostic approach.

The optimal timing for delivery in vasa previa remains a subject of ongoing discussion. The Society for Maternal-Fetal Medicine (SMFM) recommends planned cesarean delivery between 34 and 37 weeks of gestation [15]. More recent international expert consensus suggests that delivery at 36-37 weeks represents an appropriate balance between the risks of prematurity and those of membrane rupture [16]. Our decision to deliver at 36 weeks and 5 days aligned with these recommendations and the preferences of the referring physician.

Surgical considerations during cesarean delivery for vasa previa include careful uterine incision placement to avoid the aberrant vessels and preparation for possible neonatal transfusion. The surgical team should be informed of the vessel location, and O-negative blood should be immediately available for potential neonatal transfusion [17]. In our case, the careful surgical approach resulted in intact delivery without vessel rupture and excellent neonatal outcomes.

Placental pathology examination (Fig. 3, Fig. 4, Fig. 5, Fig. 6) confirmed the diagnosis and provided important documentation. The bipartite placenta demonstrated 2 distinct lobes connected by membranes containing fetal vessels—the classic morphology underlying Type II vasa previa. The velamentous cord insertion was evident, with vessels traversing the membranes before reaching placental tissue. Such pathological examination is essential for confirming the prenatal diagnosis and understanding the anatomical basis of the condition.

## Conclusion

Vasa previa remains a potentially life-threatening condition that is highly amenable to prenatal diagnosis and prevention of adverse outcomes. This case demonstrates that with appropriate identification of risk factors, targeted ultrasound screening combining grayscale and color Doppler imaging, and planned cesarean delivery before labor, excellent maternal and neonatal outcomes can be achieved. We advocate for heightened awareness of this condition among obstetric care providers and the implementation of systematic screening protocols for women with identified risk factors. The combination of prenatal ultrasound findings and postnatal placental pathology provides comprehensive documentation essential for case confirmation and education.

## Patient consent

Complete written informed consent was obtained from the patient for the publication of this study and accompanying images.
